# Postmastectomy Radiotherapy Improves Disease-Free Survival of High Risk of Locoregional Recurrence Breast Cancer Patients with T1-2 and 1 to 3 Positive Nodes

**DOI:** 10.1371/journal.pone.0119105

**Published:** 2015-03-17

**Authors:** Zhen-Yu He, San-Gang Wu, Juan Zhou, Fang-Yan Li, Qin Lin, Huan-Xin Lin, Jia-Yuan Sun

**Affiliations:** 1 Sun Yat-sen University Cancer Center, State Key Laboratory of Oncology in South China, Department of Radiation Oncology, Collaborative Innovation Center of Cancer Medicine, Guangzhou, People’s Republic of China; 2 Xiamen Cancer Center, Department of Radiation Oncology, the First Affiliated Hospital of Xiamen University, Xiamen, People’s Republic of China; 3 Xiamen Cancer Center, Department of Obstetrics and Gynecology, the First Affiliated Hospital of Xiamen University, Xiamen, People’s Republic of China; NIH, UNITED STATES

## Abstract

**Objectives:**

The indications for post-mastectomy radiotherapy (PMRT) with T1-2 breast cancer and 1-3 positive axillary lymph nodes is still controversial. The purpose of this study was to investigate the role of PMRT in T1-2 breast cancer with 1-3 positive axillary lymph node.

**Methods:**

We retrospectively reviewed the file records of 79 patients receiving PMRT and not receiving PMRT (618 patients).

**Results:**

The median follow-up was 65 months. Multivariate analysis showed that PMRT was an independent prognostic factor of locoregional recurrence-free survival (LRFS) (*P* = 0.010). Subgroup analysis of patients who did not undergo PMRT showed that pT stage, number of positive axillary lymph nodes, and molecular subtype were independent prognostic factors of LRFS. PMRT improved LRFS in the entire group (*P* = 0.005), but did not affect distant metastasis-free survival (DMFS) (*P* = 0.494), disease-free survival (DFS) (*P* = 0.215), and overall survival (OS) (*P* = 0.645). For patients without PMRT, the 5-year LRFS of low-risk patients (0–1 risk factor for locoregional recurrence) of 94.5% was significantly higher than that of high-risk patients (2-3 risk factors for locoregional recurrence) (80.9%, *P* < 0.001). PMRT improved LRFS (*P* = 0.001) and DFS (*P* = 0.027) in high-risk patients, but did not improve LRFS, DMFS, DFS, and OS in low-risk patients.

**Conclusions:**

PMRT is beneficial in patients with high risk of locoregional recurrence breast cancer patients with T1-2 and 1 to 3 positive nodes.

## Introduction

Currently, the indications for post-mastectomy radiotherapy (PMRT) are primarily based on the status of axillary lymph nodes [1–3]. However, due to the lack of appropriate randomized controlled studies, it is controversial whether patients with T1–2 breast cancer and 1–3 positive axillary lymph nodes (T1–2N1M0) require PMRT [4–6]. The 2001 American Society of Clinical Oncology (ASCO) and the 2007 Japanese Breast Cancer Society guidelines do not recommend PMRT for patients with T1–2N1M0 breast cancer [1–2].

The Danish Breast Cancer Cooperative Group (DBCG) published a study on the value of PMRT in patients with 1–3 positive axillary lymph nodes in 2007, and the results showed that the 15-year locoregional recurrence (LRR) rate declined by 41% and 23% in patients with ≥4 and 1–3 positive axillary lymph nodes, respectively, after postoperative radiotherapy and the 15-year overall survival (OS) was improved in 9% of the patients [7]. The data served as the basis on which the National Comprehensive Cancer Network (NCCN) revised the recommendation for PMRT in patients with T1–2N1M0 breast cancer to a strong recommendation after 2007 [8]. However, the DBCG study was not a randomized controlled study, and the guidelines published by the Expert Panel on Radiation Oncology (EPRO) in 2009 still did not recommend adjuvant radiotherapy in T1–2N1M0 breast cancer patients [3]. The European Society for Medical Oncology (ESMO) 2011 guidelines only recommended PMRT for patients with 1–3 metastatic axillary lymph nodes and risk factors associated with high LRR [9].

A growing body of literature supports the value of postoperative adjuvant radiotherapy in patients with T1–2N1M0 breast cancer after mastectomy [7, 10, 11], especially for patients at high risk for relapse [12–14]. At the 2013 St. Gallen Breast Cancer Conference, 64% of the experts did not recommend PMRT in patients with T1–2N1M0 breast cancer, but 62% of the experts suggested that radiotherapy should be administered to patients with adverse prognostic factors. However, the specific adverse prognostic factors were not clearly defined [15]. Therefore, identifying risk factors for LRR in patients with T1–2N1M0 breast cancer after mastectomy is a problem worthy of attention.

We retrospectively analyzed the clinical and pathological data of Chinese women with T1–2N1M0 breast cancer after mastectomy, and analyzed the prognostic factors for LRR to explore the clinical value of PMRT and to determine the subgroup of patients benefit form PMRT.

## Patients and Methods

### Patients

We retrospectively analyzed the clinical and pathological data of patients with breast cancer who were treated at the Sun Yat-Sen University Cancer Center from July 1998 to November 2007. The inclusion criteria for this analysis were: (1) Female patients with unilateral breast cancer and no distant metastasis at initial diagnosis who underwent mastectomy and axillary lymph node dissection; (2) postoperative pathology indicated T1–2 and 1–3 positive axillary lymph nodes (T1–2N1M0) disease, at least 10 lymph nodes removed by axillary dissection; (3) complete surgical resection of the tumor and negative margins; (4) complete estrogen receptor (ER), progesterone receptor (PR) and human epithelial growth factor receptor family 2 (Her2) status; (5) No neoadjuvant chemotherapy was administered before surgery and endocrine therapy was performed based on the hormone receptor status. The study was approved by the ethics committee of Sun Yat-Sen University Cancer Center. All patients provided written consent for storage of their medical information in the hospital database and for research use of this information.

### Clinicopathological factors and lymph node status

Patients clinical, pathological, and immunohistochemical data including age, menopausal status, pT stage, number of positive lymph nodes, lymph node ratio (LNR), lymphatic invasion, molecular subtype, and PMRT were used to assess the risk of LRR. LNR was defined as the ratio of the number of positive axillary lymph nodes to the number of axillary lymph nodes removed during axillary lymph node dissection. Positive ER and PR status was defined as immunohistochemical staining of at least 10% positive cells. Her2-positivity was defined as a 3+ immunohistochemical result or a 2+ immunohistochemical result confirmed by fluorescent in situ hybridization (FISH). Molecular subtypes were classified as luminal A (ER+ and/or PR+, Her2-), luminal B (ER+ and/or PR+, Her2-), Her2-enriched (ER-, PR-, and Her2+), and triple negative (ER-, PR-, and Her2-).

### Follow-up and survival endpoints

Patients were followed once every 3–6 months, with the first day after surgery as the start time of follow-up. The primary endpoint of the study was locoregional recurrence-free survival (LRFS), distant metastasis-free survival (DMFS), disease-free survival (DFS), and overall survival (OS). The survival status of patients was obtained through medical record review, phone call, or letter.

Locoregional recurrence (LRR) was defined as recurrence in the ipsilateral chest wall, supraclavicular and infraclavicular areas, axilla, and internal mammary region. The diagnosis of LRR rely mainly on physical examination, ultrasound, CT, or PET / CT, if necessary. All patients with LRR were confirmed by pathological examination. Spread of the primary cancer to sites distant from the locoregional recurrence sites were considered indicative of distant metastasis. For patients with suspected distant metastatic, bone metastasis required bone scan and MRI; lung metastasis usually was identified by repeated chest radiograph, followed by chest CT confirmation, or PET/CT confirmation; liver metastasis generally used ultrasound at follow up, and was followed by MRI or PET/ CT if an abnormality were observed, and by pathological examination when necessary. DFS referred to the presence of locoregional or distant recurrence. Overall survival (OS) was calculated as a period of time from the date of diagnosis to the date of death from any cause or the date of last follow-up.

### Statistical analysis

All data were analyzed using the SPSS statistical software package (version 16.0; IBM Corporation, Armonk, NY, USA). The χ^2^ and Fisher’s exact probability tests were used to analyze the differences between qualitative data. The survival curves were plotted by the Kaplan-Meier method and compared using the log rank test. Cox regression analysis was performed to identify significant prognostic factors. The variables with *P*-value less than 0.05 by univariate analyses were included in the multivariate analyses. A *P-*value less than 0.05 was considered statistically significant.

## Results

### Clinicopathological data and treatments

Patients data are summarized in Table 1. This study included 697 patients with a median age of 47 years (range, 23–82 years) at diagnosis. There were 662 patients (95.0%) of invasive ductal carcinoma, the median number of axillary lymph nodes removed was 15 (range, 10–42), and the median number of positive lymph nodes was 2. A total of 618 patients (88.7%) did not receive PMRT, 79 patients (11.3%) received PMRT included the ipsilateral chest wall, supraclavicular and infraclavicular region after mastectomy. The total radiation dose was 50 Gy with 2 Gy delivered over 25 times. The chest wall was treated with 6 MV X-ray with opposed tangential fields or 6–9 Mev electron beam, with the use of tissue compensation membrane of 0.5–1 cm when needed. Single-field irradiation was performed for the supra- and infra-clavicular lymph drainage regions with 6 MV X-ray combined with 12–15 Mev. Patients with age < 35 years (*P* = 0.015), pT2 stage (*P* = 0.003), three positive lymph nodes (*P* < 0.001) were more likely to undergo PMRT.

**Table 1:** Patient demographic and clinical data.

Characteristic	Entire cohort	Without PMRT (%)	With PMRT (%)	*P*
Age (y)				
<35	83	67 (10.8)	16 (20.3)	0.015
≥35	614	551 (89.2)	63 (79.7)	
Menopausal status				
Premenopausal	463	398 (64.4)	66 (83.5)	0.001
Postmenopausal	233	220 (35.6)	13 (16.5)	
Tumor size				
pT1	218	205 (33.2)	13 (16.5)	0.003
pT2	479	413 (66.8)	66 (83.5)	
Positive lymph nodes (n)				
1	334	317 (51.3)	17 (21.5)	< 0.001
2	194	175 (28.3)	19 (24.1)	
3	169	126 (20.4)	43 (54.4)	
Lymphatic invasion				
Negative	670	598 (96.8)	72 (91.1)	0.015
Positive	27	20 (3.3)	7 (8.9)	
Molecular subtype				
Luminal A	364	334 (54.0)	30 (38.0)	0.014
Luminal B	144	118 (19.1)	26 (32.9)	
Her2-enriched	101	87 (14.1)	14 (17.7)	
Triple negative	88	79 (12.8)	9 (11.4)	
LNR (%)				
≤ 20	615	562 (90.9)	53 (67.1)	< 0.001
> 20	82	56 (9.1)	26 (32.9)	
Chemotherapy				
No	30	30 (4.9)	0 (0)	0.045
Yes	667	588 (95.1)	79 (100)	

PMRT, postmastectomy radiotherapy; LNR, lymph node ratio.

Among the 667 patients (95.7%) who received adjuvant chemotherapy, an anthracycline or taxane regimen was used in 618 patients (92.7) and a cyclophosphamide, methotrexate, and 5-fluorouracil (CMF) regimen was used in 49 patients. For 24 patients with age ≥ 65 years and positive for hormone receptors, endocrine therapy alone was prescribed. Six patients with Her2-enriched or triple negative subtypes were not received adjuvant chemotherapy after mastectomy. The median number of chemotherapy cycles was 6 (range, 4–8). Five patients underwent targeted therapy with trastuzumab. All patients with positive hormone receptors underwent endocrine therapy. Premenopausal patients were treated with tamoxifen, and postmenopausal women were treated with tamoxifen or an aromatase inhibitor.

### Survival

The median follow-up duration was 65 months (range, 6–144 months), and 66 patients experienced LRR, the 5-year LRFS rate was 90.1%. LRR occurred in 65 patients who did not undergo PMRT, and in 1 patients who underwent PMRT, the 5-year LRR rates were 1.3% and 11.1% in patients with and without PMRT, respectively (*P* = 0.005). Distant metastases occurred in 127 patients, and the 5-year DMFS rate was 81.2%. The 5-year DFS rate was 78.3%. A total of 90 patients died among whom 87 died because of breast cancer and 3 died of other diseases. The 5-year OS rate was 88.1%.

### Prognostic factors for survival

**Fig 1:**
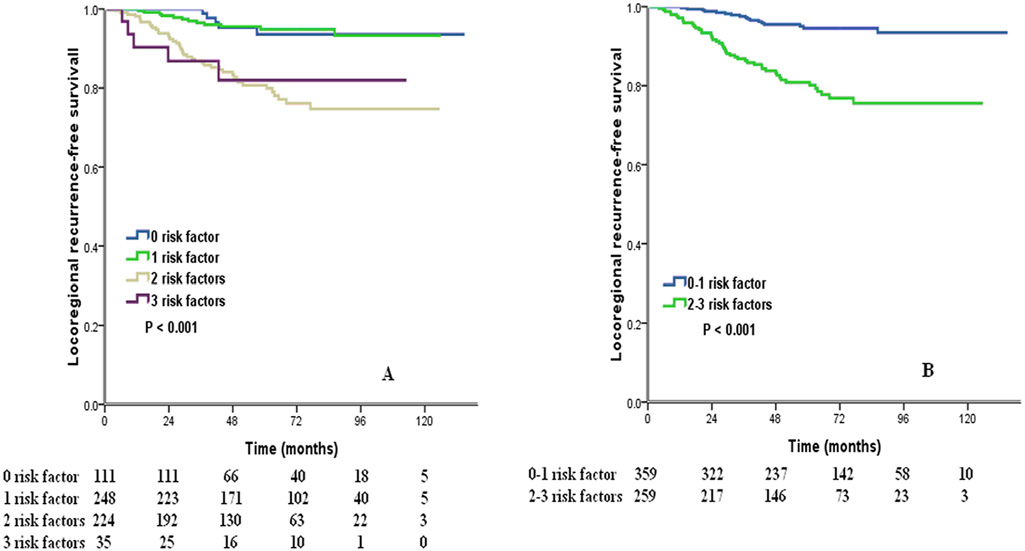
Cumulative of locoregional recurrence-free survival by risk groups (A, 4 risk factors groups; B, 0–1 risk factor vs. 2–3 risk factors).

Both univariate and multivariate analysis of all patients showed that pT stage, number of positive axillary lymph nodes, molecular subtype, and PMRT were independent prognostic risk factors of LRFS (Table 2, 3). Univariate and multivariate analysis of the patients without PMRT showed that pT stage, number of positive axillary lymph nodes, molecular subtype were independent prognostic risk factors of LRFS (Table 2, 3). For these three independent risk factors, the 5-year LRFS rates corresponding to pT2 stage, 2–3 positive axillary lymph nodes and Her2-enriched subtype were 86.8%, 84.9%, and 85.8%, respectively. The above three independent risk factors were grouped, and analysis showed that the 5-year LRFS rates corresponding to 0, 1, 2, and 3 risk factors were 93.6%, 94.9%, 80.7%, 82.0%, respectively (*P* < 0.001) (Fig. 1A). Therefore, patients with 0–1 risk factor were considered low-risk patients, and patients with 2–3 risk factors were considered high-risk patients. The 5-year LRFS rate of low-risk patients was 94.5%, which were significantly higher than those of high-risk patients (80.9%; *P* < 0.001) (Fig. 1B).

**Table 2:** Univariate analysis of prognostic factors for locoregional recurrence-free survival.

Characteristic	Entre cohort			Without PMRT		
	HR	95% CI	*P*	HR	95% CI	*P*
Age, years (<35 vs. ≥35)	1.526	0.799–2.916	0.201	1.787	0.934–3.418	0.079
Menopausal status (premenopausal vs. postmenopausal)	0.793	0.465–1.350	0.393	0.725	0.425–1.237	0.238
Tumor size (pT2 vs. pT1)	2.119	1.134–3.961	0.019	2.322	1.241–4.346	0.008
Positive lymph nodes, n (2–3 vs. 1)	1.925	1.154–3.212	0.012	2.201	1.317–3.679	0.003
Lymphatic invasion (negative vs. positive)	0.793	0.194–3.241	0.747	1.050	0.257–4.291	0.946
Breast cancer subtype						
(luminal B vs. luminal A)	1.031	0.509–2.087	0.933	1.159	0.572–2.347	0.682
(Her2 positive vs. luminal A)	2.782	1.525–5.075	0.001	2.903	1.575–5.351	0.001
(triple negative vs. luminal A)	1.899	0.938–3.844	0.075	1.966	0.971–3.978	0.060
LNR, % (> 0.20 vs. ≤ 0.20)	1.250	0.638–2.451	0.516	1.471	0.727–2.976	0.283
PMRT (yes vs. no)	0.103	0.014–0.739	0.024			

PMRT, postmastectomy radiotherapy; LNR, lymph node ratio; HR, hazard ratio; CI, confidence interval.

**Table 3:** Multivariate analysis of prognostic factors for locoregional recurrence-free survival.

Characteristic	Entire cohort			Without PMRT		
	HR	95%CI	*P*	HR	95%CI	*P*
Tumor size (pT2 vs. pT1)	2.094	1.115–3.931	0.021	2.079	1.106–3.906	0.023
Positive lymph nodes, n (2–3 vs. 1)	2.092	1.251–3.499	0.005	2.073	1.238–3.472	0.006
Breast cancer subtype						
(luminal B vs. luminal A)	1.064	0.524–2.159	0.864	1.078	0.532–2.187	0.835
(Her2 positive vs. luminal A)	2.863	1.568–5.229	0.001	2.732	1.480–5.040	0.001
(triple negative vs. luminal A)	1.929	0.952–3.907	0.068	1.936	0.956–3.922	0.067
PMRT (yes vs. no)	0.073	0.010–0.531	0.010			

PMRT, postmastectomy radiotherapy; LNR, lymph node ratio; HR, hazard ratio; CI, confidence interval.

### Effect of PMRT on survival

**Fig 2:**
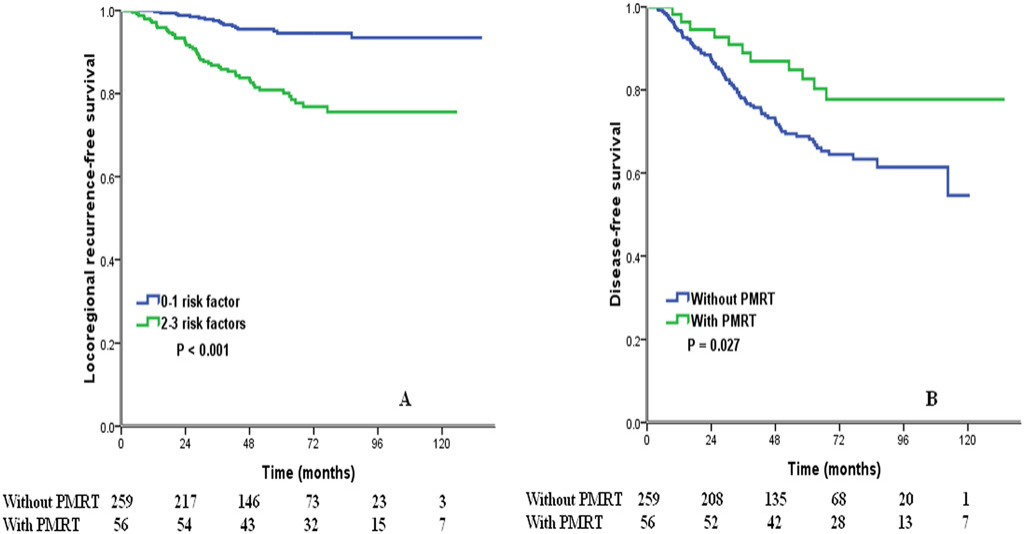
Impact of adjuvant radiotherapy on locoregional recurrence-free survival (A) and disease-free survival (B) in high-risk patients.

PMRT improved LRFS (*P* = 0.005), but did not improve DMFS (*P* = 0.494), DFS (*P* = 0.215), and OS (*P* = 0.646) in entire group. Subgroup analysis showed that for low-risk patients, PMRT did not affect LRFS (*P* = 0.265), DMFS (*P* = 0.953), DFS (*P* = 0.898) and OS (*P* = 0.328). For high-risk patients, although PMRT did not improve DMFS (*P* = 0.164) and OS (*P* = 0.444) (Table 4), it improved LRFS (*P* = 0.001, Fig. 2A) and DFS (*P* = 0.027, Fig. 2B).

**Table 4:** Effect of PMRT on survival.

Characteristic	Without PMRT (5-year)	With PMRT (5-year)	*P*
Entire cohort			
LRFS (%)	88.9	98.7	0.005
DMFS (%)	80.6	86.1	0.494
DFS (%)	77.4	84.8	0.215
OS (%)	87.3	93.1	0.646
Low-risk patients			
LRFS (%)	94.5	100	0.265
DMFS (%)	84.8	89.8	0.953
DFS (%)	83.5	89.8	0.898
OS (%)	90.0	87.5	0.328
High-risk patients			
LRFS (%)	80.9	98.2	0.001
DMFS (%)	74.8	84.5	0.164
DFS (%)	68.8	82.7	0.027
OS (%)	83.6	90.4	0.444

PMRT, postmastectomy radiotherapy; LRFS, locoregional recurrence-free survival;

DMFS, distant metastasis-free survival; DFS, disease-free survival; OS, overall survival.

## Discussion

In the present study, we examined the value of PMRT in patients with T1–2N1M0 breast cancer and found that PMRT only improved the LRFS without improving other outcomes. The subgroup analysis found that PMRT improved LRFS and DFS of patients with 2–3 risk factors for LRR (high-risk patients), while it did not improve the prognosis of patients with 0–1 risk factors for LRR (low-risk patients).

A number of studies have examined the effect of PMRT on the survival of breast cancer patients with 1–3 positive axillary lymph nodes. Cosar et al. reported that PMRT improved LRFS (*P* = 0.038), DMFS (*P* = 0.004), and DFS (*P* = 0.034) of patients with 1–3 positive axillary lymph nodes, and that it has potential benefit for OS (*P* = 0.087) [10]. However, the sample size of the study was small. The DBCG study found that PMRT improved LRFS and OS of patients with 1–3 or ≥ 4 metastatic axillary lymph nodes [7], but the results were questioned due to inadequate adjuvant therapy and insufficient number of axillary lymph nodes removed at axillary lymph node dissection. The current study included 697 patients, and median number of axillary lymph nodes removed was 15. The majority of our patients received chemotherapy and endocrine therapy, and we found that PMRT only improved LRFS without affecting other outcomes. This result is similar to the findings of Duraker et al [14].

The LRR rate is an important factor used for selecting adjuvant radiotherapy. The St. Gallen recommendations indicate that patients with a 10-year locoregional recurrence rate of 20% or more require PMRT [16]. In our study, although the 5-year LRR rate of patients who did not undergo radiotherapy was 11.1%, the subgroup analysis found that the risk of recurrence in high-risk patients was significantly higher than that of low-risk patients (19.1% vs. 5.5%, respectively, *P* < 0.001), and PMRT improved the LRFS and DFS of high-risk patients without affecting the outcome in low-risk patients. Yang et al. reported that PMRT significantly reduced the LRR rate in patients with ER negative and lymphatic invasion (40% vs. 12.5%, *P* = 0.038), and the 5-year OS also increased from 43.7% to 87.1% (*P* < 0.0001) [13]. It is obvious that PMRT can benefit patients with T1–2N1M0 breast cancer who had a high risk of LRR.

Predicting the risk of recurrence in patients at high-risk of recurrence is therefore important. The risk factors for recurrence reported by various studies vary, including pT stage, negative hormone receptor status, lymphatic invasion, Her2-positivity, number of positive lymph nodes, and LNR [12, 13, 17–20]. However, these risk factors still need to be confirmed by large-scale randomized controlled studies. Early Breast Cancer Trialists’ Collaborative Group have addressed the uncertainty of PMRT in patients with 1–3 nodes positive and concluded it reduced both recurrence and breast cancer mortality [21]. Although 86.2% of the patients received adjuvant chemotherapy in the study, but the most common chemotherapy regimen was CMF and the most common endocrine therapy was tamoxifen, the LRR and DFS rates in patients without PMRT were 21% and 55.5%, respectively [21]. In the present study, the majority of patients received adjuvant chemotherapy containing anthracycline or taxane regimen and the 5-year LRR and DFS rates were 11.1% and 77.4%, respectively. With the progress of adjuvant therapy, including anthracyclines and taxanes regimen, ovarian suppression, aromatase inhibitors, and trastuzumab, the locoregional control was better than in the past [22–25]. A recent article from the MD Anderson Cancer Center reported that in 2000–2007, 65% of the T1–2N1 patients underwent sentinel lymph node surgery, 85% were treated with taxanes, and 50% were treated with aromatase inhibitors, the 5-year LRR rates were only 4.2% and 2.8% in patients with and without PMRT (*P* = 0.48), but PMRT improved 5-year locoregional control in patients received none of above treatments in 1978–1997 (9.5% vs. 3.4%, *P* = 0.028) [26]. Thus, the benefit of PMRT in patients with T1–2 breast cancer and 1 to 3 positive lymph nodes may be decrease due to the progress of adjuvant therapy.

The randomized controlled trial Selective Use of Postoperative Radiotherapy after Mastectomy (SUPREMO) on whether T1–2N1M0 patients require PMRT, which was based on the modern adjuvant therapy. The SUPREMO has completed patient enrolling, but it will take a long time to obtain the 10-year follow-up results [5]. Since locoregional recurrence is associated with an adverse prognosis [27], the results of our study indicate that adjuvant radiotherapy should be performed in patients at high-risk of recurrence while awaiting a consensus decision on the need for postoperative adjuvant radiotherapy in T1–2N1M0 patients.

This study has some limitations. The study was a single-center retrospective study and the number of patients received PMRT was small. Although the results showed that Her2-enriched subtype was an independent prognostic factor for LRFS, most patients did not undergo trastuzumab treatment. Due to the fact that trastuzumab benefits local control in Her2+ positive patients [22], it is unclear whether targeted therapy may alter the study results.

## Conclusions

In conclusion, our results demonstrate that not all patients with T1–2N1M0 breast cancer require PMRT. PMRT is of value for patients with 2–3 risk factors for LRR (pT2 stage, 2–3 positive axillary lymph nodes, and Her2-enriched subtype).

## Supporting Information

S1 Dataset The data underlying the findings in present study. (XLS)

## References

[1] Recht A, Edge SB, Solin LJ, Robinson DS, Estabrook A, Fine RE, et al (2001) Postmastectomy radiotherapy: clinical practice guidelines of the American Society of Clinical Oncology. J Clin Oncol 19:1539–1569. 11230499 10.1200/JCO.2001.19.5.1539

[2] Japan Comprehensive Cancer Network, Breast (2007) Practice Guidelines of Surgical Therapy and Radiotherapy for Breast Cancer~A Comparison between U.S. and Japan~;.2007[2012-6-6]. Available: http://www.jccnb.net/record/images/rqe.pdf.

[3] Taylor ME, Haffty BG, Rabinovitch R, Arthur DW, Halberg FE, Strom EA, et al (2009) ACR appropriateness criteria on postmastectomy radiotherapy expert panel on radiation oncology-breast. Int J Radiat Oncol Biol Phys 73:997–1002. 10.1016/j.ijrobp.2008.10.080 19251087

[4] Nagao T, Kinoshita T, Tamura N, Hojo T, Morota M, Kagami Y. (2013) Locoregional recurrence risk factors in breast cancer patients with positive axillary lymph nodes and the impact of postmastectomy radiotherapy. Int J Clin Oncol 8:54–61. 10.1007/s10147-011-0343-y 22068463

[5] Offersen BV, Brodersen HJ, Nielsen MM, Overgaard J, Overgaard M. (2011) Should postmastectomy radiotherapy to the chest wall and regional lymph nodes be standard for patients with 1–3 positive lymph nodes? Breast Care (Basel) 6:347–351. 22619643 10.1159/000333250 PMC3357155

[6] Munshi A, Prabhu A, Kunkler I. (2012) Post mastectomy radiotherapy in one to three lymph node positive breast cancer. Acta Oncol 51:547–550. 10.3109/0284186X.2011.633929 22132746

[7] Overgaard M, Nielsen HM, Overgaard J. (2007) Is the benefit of postmastectomy irradiation limited to patients with four or more positive nodes, as recommended in international consensus reports? A subgroup analysis of the DBCG 82 b&c randomized trials. Radiother Oncol 82:247–253. 17306393 10.1016/j.radonc.2007.02.001

[8] National Comprehensive Cancer Network (NCCN) Clinical Practice Guidelines in Oncology, Breast Cancer. Version 1, 2011. 2011[2012-6-6]. Available: http://www.nccn.org/professionals/physician_gls/PDF/breast.

[9] Aebi S, Davidson T, Gruber G, Cardoso F; ESMO Guidelines Working Group. (2011) Primary breast cancer: ESMO Clinical Practice Guidelines for diagnosis, treatment and follow-up. Ann Oncol. 22(Suppl 6):vi12–24. 21908498 10.1093/annonc/mdr371

[10] Cosar R, Uzal C, Tokatli F, Denizli B, Saynak M, Turan N, et al (2011) Postmastectomy irradiation in breast in breast cancer patients with T1–2 and 1–3 positive axillary lymph nodes: is there a role for radiation therapy? Radiat Oncol 6:28 10.1186/1748-717X-6-28 21450076 PMC3072917

[11] van der Hage JA, Putter H, Bonnema J, Bartelink H, Therasse P, van de Velde CJ, et al (2003) Impact of locoregional treatment on the early-stage breast cancer patients: a retrospective analysis. Eur J Cancer 39:2192–2199. 14522378 10.1016/s0959-8049(03)00572-0

[12] Fodor J, Polgár C, Major T, Németh G. (2003) Locoregional failure 15 years after mastectomy in women with one to three positive axillary nodes with or without irradiation the significance of tumor size. Strahlenther Onkol 179:197–202. 12627264 10.1007/s00066-003-1010-7

[13] Yang PS, Chen CM, Liu MC, Jian JM, Horng CF, Liu MJ, et al (2010) Radiotherapy can decrease locoregional recurrence and increase survival in mastectomy patients with T1 to T2breast cancer and one to three positive nodes with negative estrogen receptor and positive lymphovascular invasion status. Int J Radiat Oncol Biol Phys 77:516–522. 10.1016/j.ijrobp.2009.05.016 19577858

[14] Duraker N, Demir D, Bati B, Yilmaz BD, Bati Y, Çaynak ZC, et al (2012) Survival benefit of post-mastectomy radiotherapy in breast carcinoma patients with T1–2 tumor and 1–3 axillary lymph node(s) metastasis. Jpn J Clin Oncol 42:601–608. 10.1093/jjco/hys052 22511807

[15] Klinik Onkoloji Derneği. (2013) Geographical and professional composition of the consensus panel at St Gallen 2013 [2013-6-11]. http://www.tukod.org/folders/file/Web_St_Gallen_2013.pdf

[16] Goldhirsch A, Glick JH, Gelber RD, Senn HJ. (1998) Meeting highlights: International Consensus Panel on the Treatment of Primary Breast Cancer. J Natl Cancer Inst 90:1601–1608. 9811309 10.1093/jnci/90.21.1601

[17] Matsunuma R, Oguchi M, Fujikane T, Matsuura M, Sakai T, Kimura K, et al (2012) Influence of lymphatic invasion on locoregional recurrence following mastectomy: indication for postmastectomy radiotherapy for breast cancer patients with one to three positive nodes. Int J Radiat Oncol Biol Phys 83:845–852. 10.1016/j.ijrobp.2011.08.029 22138460

[18] Wu SG, Chen Y, Sun JY, Li FY, Lin Q, Lin HX, et al (2013) Using the lymph nodal ratio to predict the risk of locoregional recurrence in lymph node-positive breast cancer patients treated with mastectomy without radiation therapy. Radiat Oncol 8:119 10.1186/1748-717X-8-119 23672513 PMC3691848

[19] Hamamoto Y, Ohsumi S, Aogi K, Shinohara S, Nakajima N, Kataoka M, et al (2014) Are there high-risk subgroups for isolated locoregional failure in patients who had T1/2 breast cancer with one to three positive lymph nodes and received mastectomy without radiotherapy? Breast Cancer 21:117–182. 10.1007/s12282-012-0369-7 22569681

[20] Wu SG, He ZY, Li FY, Wang JJ, Guo J, Lin Q, et al (2010) The clinical value of adjuvant radiotherapy in patients with early stage breast cancer with 1 to 3 positive lymph nodes after mastectomy. Chin J Cancer 29:668–676. 20591219 10.5732/cjc.009.10744

[21] Early Breast Cancer Trialists’ Collaborative Group (EBCTCG), Peto R, Davies C, Godwin J, Gray R, Pan HC, et al (2014) Effect of radiotherapy after mastectomy and axillary surgery on 10-year recurrence and 20-year breast cancer mortality: meta-analysis of individual patient data for 8135 women in 22 randomised trials. Lancet 383:2127–2135. 10.1016/S0140-6736(14)60488-8 24656685 PMC5015598

[22] Panoff JE, Hurley J, Takita C, Reis IM, Zhao W, Sujoy V, et al (2011) Risk of locoregional recurrence by receptor status in breast cancer patients receiving modern systemic therapy and post-mastectomy radiation. Breast Cancer Res Treat 128:899–906. 10.1007/s10549-011-1495-1 21475999

[23] Early Breast Cancer Trialists’ Collaborative Group (EBCTCG), Peto R, Davies C, Godwin J, Gray R, Pan HC, et al (2012) Comparisons between different polychemotherapy regimens for early breast cancer: meta-analyses of long-term outcome among 100,000 women in 123 randomised trials. Lancet 379:432–444. 10.1016/S0140-6736(11)61625-5 22152853 PMC3273723

[24] Pagani O, Regan MM, Walley BA, Fleming GF, Colleoni M, Láng I, et al (2014) Adjuvant exemestane with ovarian suppression in premenopausal breast cancer. N Engl J Med 371:107–118. 10.1056/NEJMoa1404037 24881463 PMC4175521

[25] Schiavon G, Smith IE. (2014) Status of adjuvant endocrine therapy for breast cancer. Breast Cancer Res 16:206 25032258 10.1186/bcr3636 PMC4053079

[26] McBride A, Allen P, Woodward W, Kim M, Kuerer HM, Drinka EK, et al (2014) Locoregional recurrence risk for patients with T1,2 breast cancer with 1–3 positive lymph nodes treated with mastectomy and systemic treatment. Int J Radiat Oncol Biol Phys 89:392–398. 10.1016/j.ijrobp.2014.02.013 24721590

[27] Kuo SH, Huang CS, Kuo WH, Cheng AL, Chang KJ, Chia-Hsien Cheng J. (2008) Comprehensive locoregional treatment and systemic therapy for postmastectomy isolated locoregional recurrence. Int J Radiat Oncol Biol Phys 1456–1464. 18692329 10.1016/j.ijrobp.2008.03.042

