# Correction: Postmastectomy Radiotherapy Improves Disease-Free Survival of High Risk of Locoregional Recurrence Breast Cancer Patients with T1-2 and 1 to 3 Positive Nodes

**DOI:** 10.1371/journal.pone.0145972

**Published:** 2015-12-23

**Authors:** Zhen-Yu He, San-Gang Wu, Juan Zhou, Fang-Yan Li, Qin Lin, Huan-Xin Lin, Jia-Yuan Sun

The reported study [1] is similar in aim and approach to another previously published study by some of the same authors:

“The clinical value of adjuvant radiotherapy in patients with early stage breast cancer with 1 to 3 positive lymph nodes after mastectomy,” published in the *Chinese Journal of Cancer* [2].

This previous work was cited as reference number 20 in the *PLOS ONE* paper.

In the *PLOS ONE* paper, patients receiving treatment from July 1998 to November 2007 were included in the study (79 patients received postmastectomy radiotherapy (PMRT); 618 patients did not receive PMRT). In the *Chinese Journal of Cancer* paper, patients receiving treatment from January 1998 to May 2007 were included in the study (76 patients received PMRT; 412 patients did not receive PMRT).

The authors wish to clarify the sampling methodologies. For the earlier study [2], only patients receiving both mastectomy and adjuvant therapy at Sun Yat-Sen University Cancer Center were included. The *PLOS ONE* study [1] additionally included patients who received mastectomy at other hospitals followed by adjuvant therapy at Sun Yat-Sen University Cancer Center and patients who received comprehensive therapy at other hospitals but were followed up at Sun Yat-Sen University Cancer Center. There is some overlap in patients included in the two studies, with 343 patients being included in both.

The authors also note additional differences between the two studies. The clinical value of PMRT in patients with T1-2 breast cancer and 1–3 positive axillary lymph nodes (T1-2N1M0) remained controversial following publication of the first study [2]. Therefore, in order to verify the effect of PMRT, the authors carried out the following retrospective study [1] in which the number of patients was further expanded and follow-up time was further extended. The patient selection criteria were also redefined in order to decrease the impact of other potential factors. Additional inclusion criteria were as follows: a minimum of 10 lymph nodes removed by axillary dissection; complete estrogen receptor (ER), progesterone receptor (PR) and human epithelial growth factor receptor family 2 (Her2) status known.
